# Hypertensive nonobstructive hydrocephalus as main magnetic resonance imaging feature in a dog with disseminated choroid plexus carcinomatosis

**DOI:** 10.1111/jvim.16737

**Published:** 2023-05-24

**Authors:** Lea Carisch, Lorenzo Golini, Lea Schurna, Chiara Bergamino, Katrin M. Beckmann, Monika Hilbe, Thanaporn Asawapattanakul, Wolfgang Baumgärtner, Christina Puff, Adriano Wang‐Leandro

**Affiliations:** ^1^ Clinic for Diagnostic Imaging, Department of Diagnostics and Clinical Services, Vetsuisse Faculty University of Zurich Zurich Switzerland; ^2^ Division of Neurology and Neurosurgery, Department of Small Animals Vetsuisse Faculty University of Zurich Zurich Switzerland; ^3^ Institute of Veterinary Pathology, Vetsuisse Faculty University of Zurich Zurich Switzerland; ^4^ Department of Pathology University of Veterinary Medicine Hannover, Foundation Hannover Germany; ^5^ Center for Systems Neuroscience Hannover Germany; ^6^ Department of Diagnostic Imaging, Clinic for Small Animals University of Veterinary Medicine Hannover, Foundation Hannover Germany

**Keywords:** brain imaging, canine, disseminated carcinoma, intracranial neoplasia, magnetic resonance imaging, neuroimaging

## Abstract

Obstructive or nonobstructive hypertensive hydrocephalus is reported in choroid plexus tumors. Choroid plexus tumors typically present as T2‐weighted hyperintense intraventricular masses with occasional cerebrospinal fluid‐drop metastasis. Acquired neoplastic nonobstructive hydrocephalus without visible mass lesion in magnetic resonance imaging is not reported in dogs. A 4.5‐year‐old Rhodesian Ridgeback presented with reduced mental status, unilaterally absent pupillary light reflex, and neck pain. Magnetic resonance imaging revealed a nonobstructive hydrocephalus and widened lumbar subarachnoid space with no evidence of a primary mass lesion. Postmortem examination confirmed a disseminated choroid plexus tumor affecting the ependyma and choroid plexi of all ventricles and the cerebral and lumbar subarachnoid space. Disseminated choroid plexus carcinomatosis should be considered as a possible cause of hypertensive hydrocephalus even in absence of a primary mass.

**Abbreviations**
CPC: choroidplexus carcinoma
CPT: choroidplexus tumor
CSF: cerebrospinal fluid
FLAIR: fluid attenuated inversion recovery
MRI: magnetic resonance imaging
T1W: T1‐weighted
T2W: T2‐weighted
TE: echo time
TR: time to repetition
TSE: turbo spin echo
VPS: ventriculoperitoneal shunt

## 1 INTRODUCTION

Magnetic resonance imaging (MRI) findings of choroid plexus tumors (CPT) include papilliform or globular ventricular masses, typically hyperintense on T2‐weighted (T2W) images with peritumoral edema and of variable signal intensity on T1‐weighted (T1W) imaging^1^, ^2^, ^3^, ^4^, sometimes forming cyst‐like structures.^3^, ^4^ Obstructive hydrocephalus is commonly associated to the presence of intraventricular masses obstructing cerebrospinal fluid (CSF) pathway; however, nonobstructive hydrocephalus is also postulated as possible concomitant phenomenon. Presence of ventricular system dilatation is evidenced caudal to the primary mass lesion more often in benign than malignant CPT.^4^ The choroid plexus of the fourth, the third and lateral ventricles are described as predilection sites for location of the primary tumor.^4^, ^5^, ^6^, ^7^ Signal intensity of the tumoral extension by means of meningeal carcinomatosis and/or CSF‐drop metastasis are reported as multifocal predominantly T2W hyperintense and markedly contrast‐enhancing lesions adjacent to the subarachnoid space or ventricular system.^4^, ^8^

In the present case report, we describe a dog affected by primary disseminated leptomeningeal choroid plexus carcinomatosis causing a hypertensive nonobstructive hydrocephalus as main imaging feature, without detectable primary mass over repeated MRI examinations.

## 2 CASE PRESENTATION

A 4.5 year old, 40 kg intact male Rhodesian Ridgeback was presented with an acute onset of migraine like signs,^9^ pain focused on the aural region and cervical spine, episodic pelvic limb weakness mainly when standing up and progressive reduction of activity since 21 days. The dog had a history of atopic dermatitis since puppyhood and received cyclosporine on a regular basis (Atopica, Elanco Tiergesundheit AG, Basel, Switzerland; 3.5 mg/kg, q48h).

The clinical examination revealed a body condition score 3/9. In the neurologic examination a reduced mental status, subjectively calmer demeanor, normal gait, low carried head posture, mild orthostatic tremor in the pelvic limbs and reduced proprioception of both hind limbs were detected. The pupils were mildly anisocoric with mild mydriasis in the left eye as well as absent menace response and pupillary light reflex on the left. Therefore, the neuroanatomical localization was defined multifocal intracranial and lumbar spinal cord. In the ophthamoligic examination there was severe bilateral papilledema accentuated on the left side consistent with increased intracranial pressure.

Blood chemistry panel and hematology revealed mild monocytosis (1.03 × E3/μL; reference value 0.2‐0.92 × 10E3/μL), mild hyperalbuminemia (41 g/L; reference value 29‐37 g/L), and mildly decreased blood nitrogen (2.8 mmol/L; reference value 3.8‐9.4 mmol/L).

MRI of the brain and lumbar spine was performed (Table S1) under general anesthesia using a 3 Tesla magnet (Philips Ingenia, Philips AG, Zurich, Switzerland).

Post‐contrast T1W sequences were acquired after intravenous administration of gadolinium‐based contrast agent (Omniscan, GE Healthcare AG, Opfikon, Switzerland; 0.1 mmol/kg).

**FIGURE 1:**
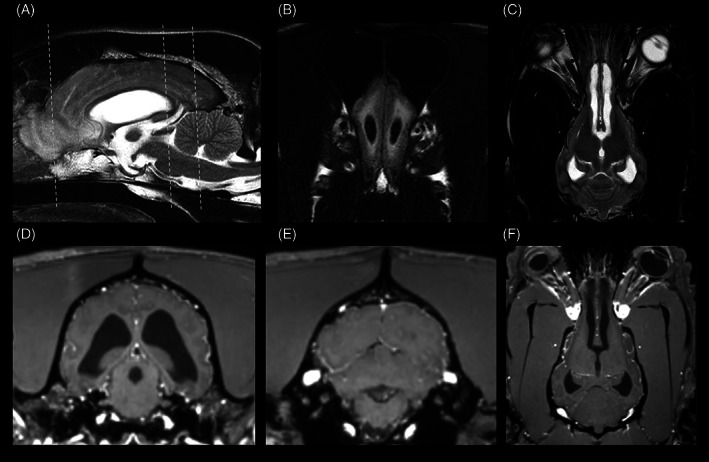
Sagittal T2W sequence showing moderate dilatation of the lateral and third ventricles and reduced diameter and oval‐shaped thalamic adhesion (A). Transverse FLAIR sequence at level of the olfactory bulbs, indicated by the white dashed line in (A), showing a moderate dilatation of the olfactory recesses and adjacent FLAIR hyperintensities, compatible with transependymal interstitial edema (B). Dorsal T2W sequence showing moderate dilatation of the olfactory recesses (C). Transverse (D + E, indicated by the white dashed lines in A) and dorsal (F) T1W post‐contrast sequence at the level of the mesencephalic aqueduct (D), lateral recesses of the 4th ventricle (E) and Monro foramina (F), showing moderate dilatation of the mesencephalic aqueduct and the Monro foramina. Furthermore, no signs of obstruction can be identified. Images displayed in radiological convention: right side of the dog is to the left of the image, rostral is to the left of the image.

MRI of the brain revealed a moderate lateral and third ventriculomegaly with moderate distention of the mesencephalic aqueduct (Figure 1A‐F). Further, an ill‐defined reduced signal intensity of the CSF at the mesencephalic aqueduct was present in sagittal T2W images only and interpreted as possible CSF flow‐related artifact. The interthalamic adhesion was reduced in diameter and oval shaped, compatible with increased intraventricular pressure (Figure 1A). The cerebral cortical gyri were flattened, with compression of the sulci, and the olfactory recesses were moderately dilated bilaterally, with severe peripheral fluid attenuated inversion recovery (FLAIR) hyperintensities accentuated at the white matter and extending caudally adjacent to the lateral ventricles up to the level of the optic canals, compatible with trans‐ependymal interstitial periventricular edema. Additionally, the septum pellucidum could not be completely visualized and a mild protrusion of left optic disc into the vitreous body was present, compatible with papilledema. After contrast‐medium administration, no areas of increased intra‐ or extraaxial enhancement were detected.

**FIGURE 2:**
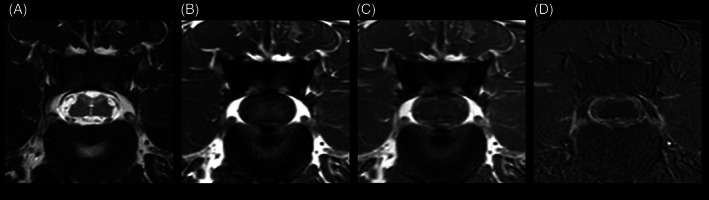
Transverse T2W (A), T1W (B), T1W post‐contrast (C), and subtraction (D) images of the vertebral column at the level of L4‐5 showing a severely widened subarachnoid space with moderate compression and deformation of the spinal cord, as well as symmetrical indentations. An increased T2 signal intensity in the region of the dorsal horns and dorsal funiculus and dilated central canal can be seen in (A). Marked contrast enhancement of the meninges and subarachnoid space is visible in (C) and (D). Images displayed in radiological convention: right side of the dog is to the left of the image.

At the level of the lumbar intumescence, the subarachnoid space was circumferentially widened, compressing the spinal cord, which showed symmetrical indentations associated with the septum posticum and denticulate ligaments. The central canal was moderately dilated, and the dorsal horns and dorsal funiculus mildly and diffusely increased in T2W signal intensity. After contrast‐medium administration, marked enhancement of the meninges and subarachnoid space was present, most conspicuous in subtraction images (Figure 2A‐D).

Based on these findings, imaging diagnosis was a hypertensive nonobstructive hydrocephalus and severe lumbar meningomyelopathy with circumferential intradural extramedullary compression of the lumbar intumescence because of dilatation of the subarachnoid space.

During cisterna magna tap, CSF pressure was measured—using a Compact Modular Patient Monitor (Carescape B650, GE healthcare by anandic, Helsinki, Finland). An increase of CSF pressure at the cisterna magna was measured in lateral recumbency (37 mm Hg: expected range 7.6‐15.2 mm Hg^10^). Cerebrospinal fluid analysis yielded a mild mononuclear pleocytosis (leucocytes 8.7 cells/μL, 70% monocytes, 30% lymphocytes; reference value: <5 cells/μL) and a total protein of 48 mg/dL (reference value: 30 mg/dL). IgM and IgG for tick‐borne encephalitis measured in CSF were negative; PCR for *Neospora caninum*, canine distemper virus and toxoplasmosis yielded also negative results.

A clinical diagnosis of hypertensive hydrocephalus with flow disturbance and suspected noninfectious meningomyelitis was made. The dog was discharged with pain management (paracetamol [Paracetamol Mepha, Mepha Pharma AG, Basel, Switzerland, 10 mg/kg q8h] and gabapentin [Gabapentin Mepha, Mepha Pharma AG, Basel, Switzerland, 10 mg/kg q8h] as well as prednisolone [Prednisolon Streuli, Streuli Pharma AG, Uznach, Switzerland, 0.75 mg/kg q24h]); the administration of cyclosporine was stopped.

Twenty‐one days later, the dog was presented again because of generalized, tonic‐clonic seizures. A ventriculoperitoneal shunt (VPS) was placed within the left lateral ventricle. A post‐surgical MRI of the brain was performed to document the correct placement of the VPS and reevaluate the previously described pathological findings, which remained unchanged.

The dog was discharged in good general condition 3 days after surgery with instructions to administer pregabalin (Pregabalin Mepha, Mepha Pharma AG, Basel, Switzerland, 5 mg/kg q8h), levetiracetam (Levetiracetam Mepha, Mepha Pharma AG, Basel, Switzerland, 20 mg/kg q8h) and prednisolone (Prednisolon Streuli, Streuli Pharma AG, Uznach, Switzerland, 0.3 mg/kg q24h).

Forty‐nine days later, the dog was presented because of severe apathy and moderate inappetence, hyperthermia, shivering and moderate dehydration. Bacterial culture from the shunt implant site determined the presence of *Staphylococcus pseudintermedius* and systemic antibiotic therapy with clindamycin (Clindamycin Phosphat Pfizer, Pfizer AG, Zurich, Switzerland, 11 mg/kg q8h) and marbofloxacin (Marbocyl FD ad.us. vet., Vetoquinol AG, Bern, Switzerland, 2 mg/kg q24h) was started according to antibiogram. The VPS was removed, and a negative pressure drain system (KCI V.A.C Therapy System, San Antonio, Texas, USA) was placed at the wound level. The transparent, CSF‐like fluid drained from the surgical site was measured daily for the following 4 days post‐surgery, with an average volume of 300 mL/day. After 4 days, the drain system was removed, and a new VPS was surgically placed within the contralateral ventricle.

The dog was re‐examined every 21 days. No abnormalities were detected on clinical and neurological examination, and the owner reported a subjective full recovery.

The dog was presented to the emergency service 112 days after initial presentation because of focal facial seizures, disorientation, circling, hyperthermia, pain within the neck region, and hypersalivation.

**FIGURE 3:**
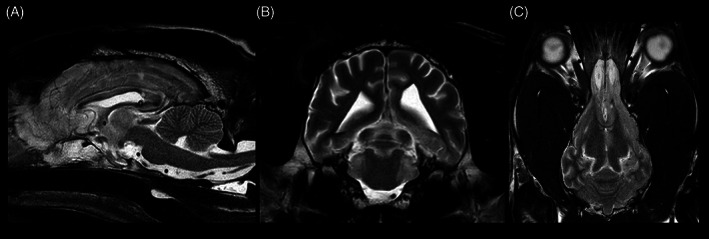
Sagittal (A), transverse (B), and dorsal (C) T2W sequence showing regression of the hydrocephalus compared to the state before the shunt placement. Images displayed in radiological convention: right side of the dog is to the left of the image, rostral is to the left of the image.

**FIGURE 4:**
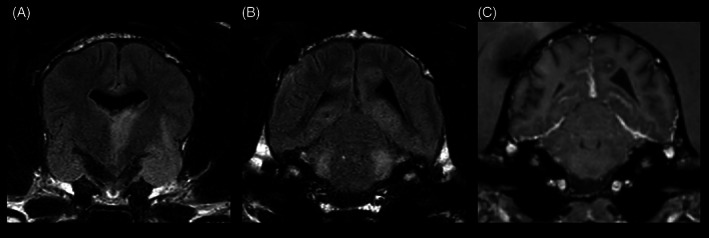
Transverse FLAIR sequence showing hyperintense intraaxial lesions at the left thalamus and piriform lobes (A) and pons, cingulate gyri and parahippocampal gyri (B) as well as marked leptomeningeal enhancement at the region of the pons in a transverse T1W post‐contrast sequence (C). Images displayed in radiological convention: right side of the dog is to the left of the image.

A second MRI‐study was performed, using the same protocol as the initial scan (Table S1). The VPS was in situ. A regression of the diagnosed hydrocephalus with a mild ventricular asymmetry was evident (Figure 3A‐C). Additionally, multifocal, T2W, and FLAIR hyperintense intraaxial lesions within the left thalamic region, piriform lobes, cingulate gyri, parahippocampal gyri, and pons were present (Figure 4A‐C). After contrast‐medium injection, a marked, diffuse, extensive, ventrally distributed leptomeningeal enhancement was present.

Cerebrospinal fluid analysis revealed minimal monocytic pleocytosis (6 cells/μL; reference value: <5 cells/μL) with increased total protein (47 mg/dL; reference value: 30 mg/dL), but no infectious agents or neoplastic cells were detected.

Owners reported no clinical improvement under ongoing treatment with antiseizure medications (phenobarbital [Aphenylbarbit, Streuli Pharma AG, Uznach, Switzerland, 3 mg/kg BID]; levetiracetam [Levetiracetam Mepha, Mepha Pharma AG, Basel, Switzerland, 20 mg/kg/q8h]), anti‐inflammatory drugs (prednisolone [Prednisolon Streuli, Streuli Pharma AG, Uznach, Switzerland, 0.25 mg/kg/q48h, reduced to 0.125 mg/kg/q48h]) and antibiotics (marbofloxacin [Marbocyl ad. us. vet., Vetoquinol AG, Bern, Switzerland, 20 mg/kg/q24h]). A sudden deterioration of forebrain signs occurred 154 days after initial presentation and the owner elected for euthanasia and necropsy.

**FIGURE 5:**
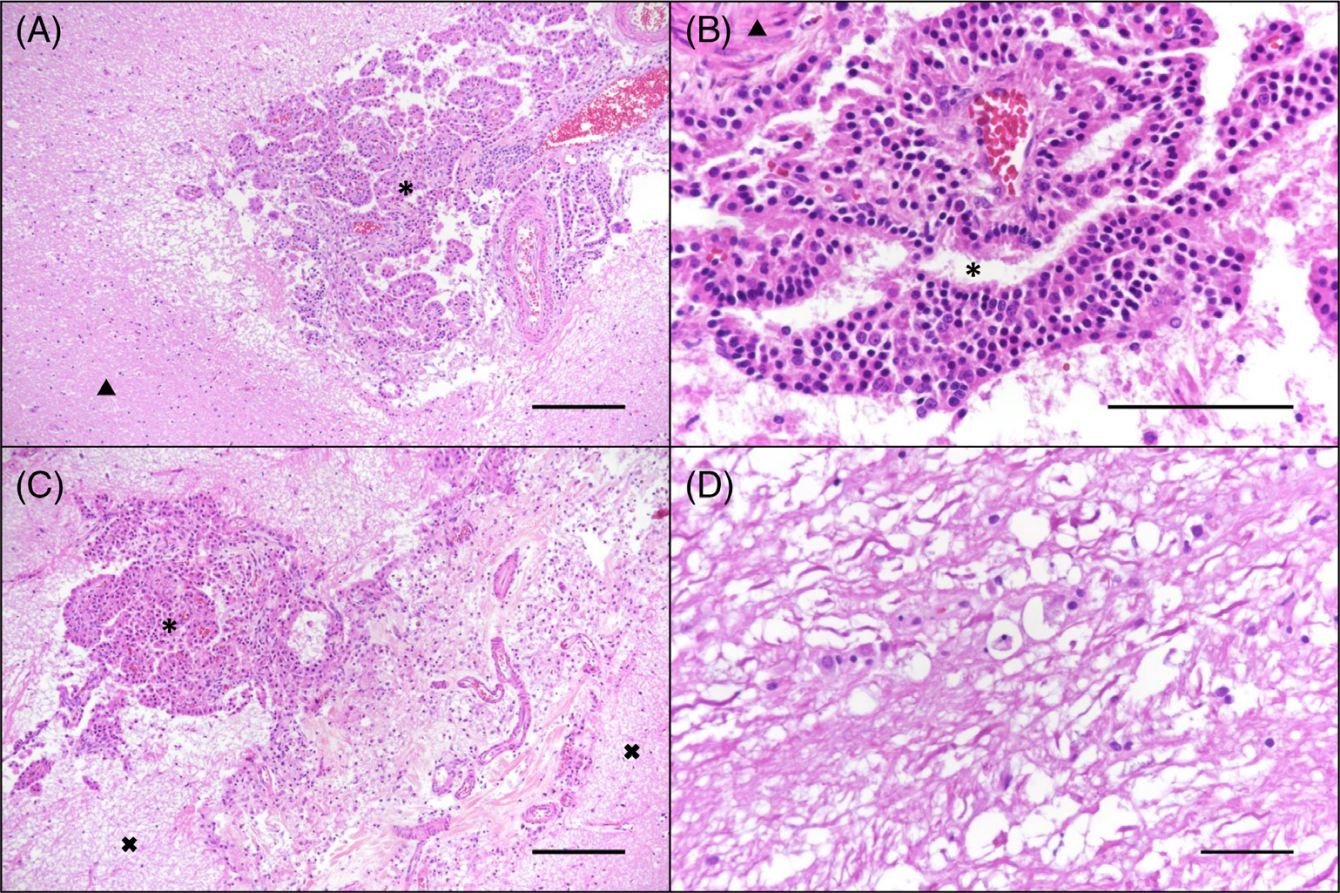
Brain and spinal cord, Hematoxylin and eosin stain, bar indicates 200 μm in A‐C and 50 μm in D. * indicating the ventricular space, △ indicating adjacent brain parenchyma, x indicating adjacent spinal cord parenchyma. (A) Region of Ammon's horn: unencapsulated, invasive growth of neoplastic cells with moderate vacuolization and mild gliosis within the adjacent neuropil. (B) Unencapsulated mass between cerebellum and cerebrum: round to columnar neoplastic cells with well‐defined cell borders and moderately abundant eosinophilic cytoplasm. (C) Lumbar spinal cord: invasive growth of the mass within the subarachnoidal space. (D) Lumbar spinal cord: activated astrocytes and myelinophages in dilated myelin sheaths.

**FIGURE 6:**
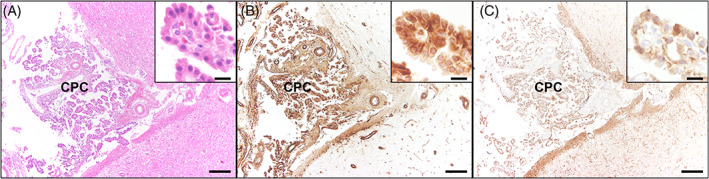
Brain, Region of Ammon's horn. Hematoxylin and eosin stain (A), immunhistology (B + C), bar indicating 200 μm, insert bar 15 μm. (A) Morphological overview of the choroid plexus carcinoma (CPC) in the brain of the dog depicting multiple plump papillary projections (insert, higher magnification). (B) The majority of neoplastic cells within the CPC expressed vimentin (insert, higher magnification). (C) Multifocally, the tumor cells were immunolabeled by pan‐cytokeratin (insert, higher magnification).

At post‐mortem examination, macroscopically the brain showed only a moderate dilatation of the olfactory recesses. No abnormalities of the spinal cord were detected macroscopically. Microscopic findings of the brain revealed an invasively growing, unencapsulated neoplasia within all ventricles and in the cerebral subarachnoid space with low cellularity and papillary structure with multifocal multilayered papillae. The neoplastic cells were round to columnar with well‐defined cell borders and moderately abundant eosinophilic cytoplasm. The nuclei were round, basally located with indistinct nucleoli. Mild anisocytosis and anisokaryosis were present, whereas no mitoses were detected. The adjacent neuropil showed decreased eosinophilia, moderate vacuolization, and mild gliosis within the gray and white matter, as well as activated astrocytes and myelinophages in dilated myelin sheaths (Figure 5A‐D). Furthermore, mild lymphoplasmacytic encephalitis surrounding the changes described above, as well as mildly dilated lateral ventricles (hydrocephalus internus) were noted. Similar histopathological changes were found within the subarachnoid space at the level of L4‐L5 spinal cord segments. Immunohistology of the brain showed that the majority of the neoplastic cells expressed vimentin. Multifocally, the neoplastic cells were immunolabeled by pan‐cytokeratin (Figure 6A‐C).

Findings were consistent with a diffuse form of choroid plexus carcinoma (CPC) with extensive meningeal and subarachnoidal spreading without evidence of a primary mass.

## 3 DISCUSSION

CPT are described extensively in veterinary literature. Typical MRI features of CPT in dogs include solitary ventricular masses displaying increased T2W and unchanged to decreased T1W signal intensity relative to gray matter, most commonly within the fourth, third or lateral ventricle.^1^, ^4^ In post‐contrast T1W images, CPTs enhance markedly.^4^, ^11^ Although presence of CSF‐drop metastases can occur in up to 19% of dogs with CPT,^4^, ^8^ in the vast majority they are secondary to a primary ventricular mass lesion. Imaging findings of diffuse carcinomatosis without clear presence of primary mass is described in a single dog; however, in that case, multifocal, marked intraaxial cystic lesions were found.^3^ Another case report describes subdural fluid accumulation, as well as intraaxial cyst like lesions as features of a CPC in 1 dog.^12^ The present case report describes an atypical form of disseminated choroid plexus carcinomatosis with extensive meningeal spread without evidence of a primary mass. Main MRI findings included nonobstructive hydrocephalus and left papilledema, as well as circumferential intradural, extramedullary compression of the lumbar intumescence. MRI of the cervical spine was not performed caudal to C3 therefore an obstructive lesion could have been missed at this stage.

Although the diffuse carcinomatosis was identified as etiology in histopathology, a clear pathophysiologic mechanism for the nonobstructive hydrocephalus could not be identified neither in MRI nor in histopathology. Hypertensive, nonobstructive hydrocephalus can occur because of impaired CSF absorption, such as in aquaporin dysregulation or villous atrophy, or because of overproduction.^4^, ^13^, ^14^ In CPT, increased production of CSF because of hypersecretory neoplastic cells has been postulated as a possible cause or contributing factor to the hydrocephalus formation.^15^, ^16^, ^17^, ^18^

Up to 300 mL/day of CSF‐like fluid were removed from VPS site in the presented case. Although we cannot state that the drained fluid is uniquely CSF with certainty, because of the transparent macroscopic appearance and amount, we speculate that most of it would have been CSF. If so, this would have been considered a highly increased CSF production, as the reported CSF production rate in the dog is approximately 68 mL/day^19^; a mild amount of superficial wound secretion adding to the measured drained volume cannot be ruled out. Therefore, a CSF overproduction or a combination of overproduction and reduced absorption are speculated to be more likely mechanisms than reduced absorption alone. Furthermore, the concomitant meningoencephalitis in the later stages of disease course could have contributed to the persistence of the hydrocephalus. As described in literature, sterile or infectious inflammatory processes can lead to leptomeningeal protein precipitation or deposition of blood clots or fibrotic changes of the arachnoid villi, impairing therefore the CSF absorption.^4^, ^13^, ^20^

The dog received long‐term cyclosporine for treatment of atopic dermatitis since puppyhood. In human medicine, cyclosporine is reported as a rare cause of intracranial hypertension, leading to secondary medication induced papilledema.^15^ The pathomechanism of cyclosporine‐induced hypertensive hydrocephalus in humans remains unclear.^21^ Therapy discontinuation in such cases leads to clinical resolution.^21^, ^22^, ^23^, ^24^ Although not described in veterinary literature, an empiric clinical decision of discontinuing cyclosporine was made after the first MRI scan. No improvement was observed after cyclosporine discontinuation.

Long‐term administration of cyclosporine increases the risk for bacterial and fungal infections in dogs and might well contribute to the shunt infection in this case.^25^, ^26^, ^27^, ^28^ Further, long‐term administration of cyclosporine is associated with increased risk for development of neoplasia in dogs and humans.^29^, ^30^ However, CPTs have are linked to cyclosporine treatment in humans and dogs.

The papilledema was interpreted as a feature of increased intracranial pressure. However generalized microvasculopathy after long‐term administration of cyclosporine is a possible contributing factor to hypertension^31^ or optic neuropathy and optic disc swelling.^24^, ^32^ The presence of papilledema was identified bilaterally and asymmetrically in the ophthalmologic examination and only on the left side in the MRI examination. Targeted imaging of the optic nerve and disc are challenging to achieve in a field of view, slice thickness and reconstruction matrix intended for whole brain imaging. Spatial resolution and partial volume effects play an important role in identification of such small structures and might explain the abovementioned mismatch between ophthalmologic examination and MRI. Microcoils for ocular imaging could have been used to address these concerns.^33^

The CSF analysis revealed a mild pleocytosis and a marked increase in protein concentration. Together with imaging findings this was interpreted as a nonspecific finding, likely related with impaired CSF flow.^34^ Even though several studies describe exfoliation of cancer cells and spread through the ventricular and subarachnoid space^6^ the sensitivity for detection of carcinomatous cells within the CSF is low,^1^ with detection in only approximately 50% of the analyzed samples.^4^ It is not uncommon, that MRI and CSF analysis do not lead to a definitive diagnosis and biopsy samples might be indicated for a confirmed diagnosis. Surgical sampling of the meninges at the lumbar intumescence, after the first MRI, could have been performed for further diagnostic workup and might have yielded to a definitive diagnosis. Instead, a symptomatic treatment was prioritized, as a primary diffuse meningeal neoplasia was not considered as highly likely.

In conclusion, diffuse choroid plexus carcinomatosis can occur in the absence of a primary mass, mimicking inflammatory disease and causing nonobstructive hypertensive hydrocephalus.

## CONFLICT OF INTEREST DECLARATION

Authors declare no conflict of interest.

## OFF‐LABEL ANTIMICROBIAL DECLARATION

Authors declare no off‐label use of antimicrobials.

## INSTITUTIONAL ANIMAL CARE AND USE COMMITTEE (IACUC) OR OTHER APPROVAL DECLARATION

Authors declare no IACUC or other approval was needed.

## HUMAN ETHICS APPROVAL DECLARATION

Authors declare human ethics approval was not needed for this study.

## Supporting information


**Table S1:** MRI protocol parameters. Acquired pre‐ and post‐ contrast medium administration.
