# Supplementary material for: Hypertensive nonobstructive hydrocephalus as main magnetic resonance imaging feature in a dog with disseminated choroid plexus carcinomatosis
Source: J Vet Intern Med. 2023 May 24;37(4):1493–500. doi: 10.1111/jvim.16737 (PMC10365043; doi:10.1111/jvim.16737)
Supplement: Supplementary file 1 — Table S1: MRI protocol parameters. Acquired pre‐ and post‐ contrast medium administration. [file JVIM-37-1493-s001.pdf]

Supplemental Table 1: MRI protocol parameters. Acquired pre- and post- contrast medium administration.

Location	Sequence	Plane	TE (ms)	TR (ms)	ST (mm)
Brain	T2W-TSE	transverse	5597.03	100	3
	T2W-TSE	sagittal	2570.97	100	3
	T2W- TSE	dorsal	3374.35	100	2.5
	T1W	transverse	9.99	4.62	0.9
	FLAIR	transverse	11000	125	3
	3D T1W*	sagittal	9.99	4.62	0.8
Lumbar Spine	T2W	transverse	2073.09	100	3
	T2W	sagittal	3454.15	100	2.5
	T1W TSE*	transverse	572.61	8	3

T1W: T1-weighted; T2W: T2-weighted; FLAIR: fluid attenuating inversion recovery; TSE: turbo spin echo; TE: echo time; TR: repetition time; ST: slice thickness.
